# Implication of Ventricular Assist Devices in Extracorporeal Membranous Oxygenation Patients Listed for Heart Transplantation

**DOI:** 10.3390/jcm8050572

**Published:** 2019-04-26

**Authors:** Ashrith Guha, Bashar Hannawi, Ana S. Cruz-Solbes, Duc T. Nguyen, Brian A. Bruckner, Barry Trachtenberg, Edward A. Graviss, Arvind Bhimaraj, Myung Park, Imad Hussain, Thomas E. MacGillivray, Erik E. Suarez, Jerry D. Estep

**Affiliations:** 1 Department of Cardiology, Houston Methodist J.C. Walter Transplant Center, Houston Methodist Hospital, 6550 Fannin St., Houston, TX 77030, USA; babruckner@houstonmethodist.org (B.A.B.); btrachtenberg@houstonmethodist.org (B.T.); abhimaraj@houstonmethodist.org (A.B.); parkmh0519@gmail.com (M.P.); ihussain@houstonmethodist.org (I.H.); eesuarez@houstonmethodist.org (E.E.S.); 2 Houston Methodist DeBakey Heart & Vascular Center, Houston Methodist Hospital, 6550 Fannin St, Houston, TX 77030, USA; basharhannawi@gmail.com (B.H.); sofiacruzs@gmail.com (A.S.C.-S.); tmacgillivray@houstonmethodist.org (T.E.M.); 3 Houston Methodist Research Institute, Department of Pathology and Genomic Medicine, 6670 Bertner Ave, Houston, TX 77030, USA; dtnguyen6@houstonmethodist.org (D.T.N.); eagraviss@houstonmethodist.org (E.A.G.); 4 Department of Cardiovascular Medicine, Cleveland Clinic, 9500 Euclid Avenue, Cleveland, OH 44195, USA; estepj@ccf.org; 5 Heart and Vascular Institute, Kaufman Center for Heart Failure, Cleveland Clinic, 9500 Euclid Avenue, Cleveland, OH 44195, USA

**Keywords:** ventricular assist devices, heart transplant, veno-arterial extracorporeal membranous oxygenation, organ allocation

## Abstract

The new allocation criteria classify patients on veno-arterial extracorporeal membranous oxygenation (VA-ECMO) as the highest priority for receiving orthotopic heart transplantation (OHT) especially if they are considered not candidates for ventricular assist devices. The outcomes of patients who receive ventricular assist devices (VADs) after being listed for heart transplantation with VA-ECMO is unknown. We analyzed 355 patients listed for OHT with VA-ECMO from the United Network for Organ Sharing database from 2006 to 2014. Univariate and multivariate Cox proportional-hazards models were used to determine the contribution of prognostic variables to the outcome. Thirty-three patients (9.3%) received VADs (15 dischargeable, 7 non-dischargeable VADs). The VAD and non-VAD groups had similar listing characteristics except that the VAD group were more likely to have non-ischemic cardiomyopathy (48.5% vs. 25.2%), and less likely to be obese (6.1% vs. 25.2%) or have a history of prior organ transplant (3% vs. 31.1%). Patients who underwent VAD implantation had more days on the list (median 189 vs. 14 days) compared to the non-VAD group. Amongst the patients who had VADs, (25/33) 75.5% patients were subsequently transplanted with similar post-transplant survival compared to the non-VAD group (72% vs. 60.5%; *p* = 0.276). Predictors of one-year post-transplant mortality included panel reactive antibodies (PRA) class I ≥ 20%, recipient smoking history, increased serum creatinine and total bilirubin. Therefore, a small proportion of patients listed for transplantation with VA ECMO undergo VAD implantation. Their waitlist survival is better than non-VAD group but with similar post-transplant survival.

## 1. Introduction

Patients with refractory cardiogenic shock have a poor prognosis despite durable ventricle assist device (VAD) [1,2], or orthotopic heart transplant (OHT) [3]. Veno-arterial extra corporeal membrane oxygenation (VA-ECMO) can be a lifesaving intervention for these patients. While a minority of these patients recover their cardiac function to facilitate VA-ECMO wean and removal, a sizeable majority will still need to be bridged to a definitive therapy which often is OHT. The new organ allocation criteria give patients supported by VA-ECMO the highest priority (status 1) for receiving OHT, due to the fact that patients listed on VA-ECMO have the highest mortality while waiting for OHT [4]. It also requires that for the continuation of highest priority status patients not be candidates for durable mechanical support. It is, however, unclear if implantation of any adult (dischargeable or non-dischargeable) ventricular assist device (VAD) in these patients is associated with better survival on the waitlist or after transplantation. We analyzed the UNOS (United Network for Organ Sharing) database to identify patients who underwent VAD implantation after being listed with VA-ECMO and studied the impact of VAD implantation on outcomes in this population. 

## 2. Materials and Methods

### 2.1. Study Population

The study used a subset of the UNOS Standard Transplant Analysis and Research (STAR) file, which includes de-identified clinical data on all patients listed for OHT from 1 January, 2006 to 31 December, 2014. The United Network for Organ Sharing (UNOS) registry contains no patient identifiers and is publicly available. This study, therefore, qualifies as exempt from our Institutional Review Board. Standard transplant research files with follow-up data were provided by the UNOS database. The interpretation and reporting of these data are the responsibility of the author(s) and in no way should be seen as an official policy of or interpretation by the OPTN or the U.S. Government.

The UNOS database is internally linked to the Social Security Death Master File for assessment of survival once a listed patient is removed from the heart transplant waiting list. More than 98% of deaths are recorded in this file within three months from death [5,6]. The data reported here have been supplied by the United Network for Organ Sharing as the contractor for the Organ Procurement and Transplantation Network. For the current analysis, we included patients who were listed for heart transplant aged 18 years and older and who had been on VA-ECMO at the time of listing. We excluded patients undergoing heart-lung transplantation and planned multi-organ transplantation. We defined VAD as any dischargeable or non-dischargeable VAD. Durable VADs were defined in accord with OPTN published policy [7]. The non-dischargeable VADs included the AbioMed devices in agreement with the OPTN/UNOS list of percutaneous and surgically placed non-dischargeable devices. We also excluded patients with ongoing VAD support prior to listing with VA-ECMO.

### 2.2. Study Design

After patients were listed on VA-ECMO, they had four different outcomes: OHT, VAD implantation, death, or survival at one-year post-listing without the need for OHT or VAD implantation. All patients were followed for one year post-listing. Transplanted patients were followed for one year post-transplant. First, we studied predictors of waitlist mortality defined as any death within one year from listing prior to transplant (outcome censored for OHT) according to the Scientific Registry of Transplant Recipients (SRTR). Second, we studied the impact of VA-ECMO on one-year post-OHT survival and the predictors of this survival. Patients who were transplanted were then separated into two groups and compared: (a) non-VAD group, i.e., patients transplanted directly from VA-ECMO, and (b) VAD group, i.e., patients transplanted from VAD after a period of initial support by VA-ECMO.

Data collected included: patient demographics, primary diagnosis at the time of listing (coronary artery disease, ischemic, non-ischemic and restrictive cardiomyopathy), history of previous organ transplant, cerebrovascular disease, smoking, diabetes, malignancies, and prior cardiac surgery, serum creatinine and bilirubin levels, estimated glomerular filtration rate (eGFR), dialysis and mechanical ventilation, and days from listing to transplant. Hemodynamics data measured at the time of transplant included: mean pulmonary artery pressure, mean pulmonary capillary wedge pressure, and cardiac output. Transplant-specific data included: recipient and donor-specific data, such as panel reactive antibody (PRA) class, ischemic time, cytomegalovirus (CMV) status, donor-recipient gender mismatch, donor’s age, body mass index (BMI), and donor history of diabetes, hypertension and smoking. There was a small number of missing data in a few variables: previous malignancy (6/355, 1.7%), serum creatinine (4/355, 1.1%), and BMI (1/355, 0.3%).

### 2.3. Statistical Analysis

Demographic and clinical data were reported as median and interquartile range (IQR) for continuous variables, and as frequencies and proportions for categorical variables. Differences between groups were compared using a chi-square test for categorical variables and the Kruskal-Wallis test for continuous variables. Univariate and multivariate Cox proportional-hazards models were used to identify risk factors for mortality on the waiting list and after OHT. The selection for variables for the multivariate Cox proportional hazards models was conducted using the Bayesian model averaging (BMA) method [8,9]. Briefly, all variables having a *p*-value of <0.2 in the univariate analysis or variables deemed as clinically important were included in the initial multivariate model. The Stata’s BMA program was run using the variables of the initial multivariate model and suggested possibly good models which included the variables with a high probability of being a risk factor. The likelihood ratio test was used to further reduce model subsets. The best model was selected based on the small Bayesian information criterion (BIC). The performance of the predictive models was determined by calculating the Harrell’s c statistic. All the analyses were performed on Stata version 14.2 (StataCorp LLC, College Station, TX, USA). A *p*-value of <0.05 was considered statistically significant. Cox proportional hazards models were conducted based on the patients who have complete data for all variables. The Cox model for waitlist mortality excluded only one patient with missing BMI. Whereas, the Cox model for one-year survival after transplant excluded 16 patients with missing data (PRA 7.1%, bilirubin 3.3%, and creatinine 1.3%). Little’s chi-square test assessing for missing completely at random (MCAR) and covariate-dependent missingness (CDM) had a *p*-value of 0.79 and 0.95, respectively, suggesting that the missing data were completely at random and the missing data did not influence the outcome. To better understand the ideal timing when VAD implantation should be considered, competing outcomes of death, transplant, and recovery were estimated from the cohort after excluding patients who underwent VAD implantation. The changes by day after listing in the cumulative incidence, as well as slope of the competing risk curves, were examined to approximate the time-point(s) when the patient had the largest rate of change in the risk of death or recovery. Competing outcomes of death, transplant, and recovery were estimated using the method of Fine and Gray [10].

## 3. Results

### 3.1. Baseline Characteristics and Outcomes

A total of 27,106 patients were listed for OHT between January 2006 and December 2014. Of these patients, 414 (1.5%) were listed on VA-ECMO. After excluding patients with VAD support prior to VA-ECMO (*n* = 59), 355 patients were included in the final analysis. The median age of the cohort was 50 years old. Most patients were male and Caucasian (66.5% and 83.7%, respectively). 23.5% of patients were obese (BMI ≥ 30kg/m^2^) and 27.3% had non-ischemic cardiomyopathy.

**Figure 1:**
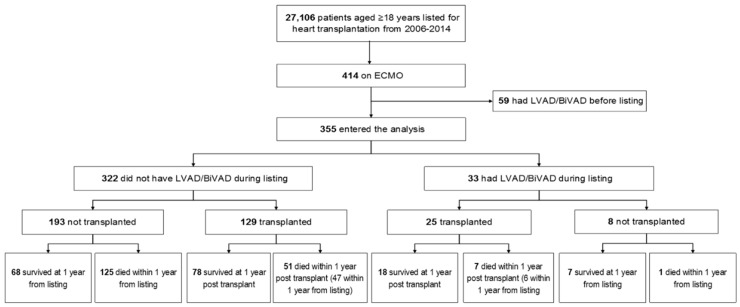
Study algorithm showing outcomes of patients listed on VA-ECMO. VA-ECMO: veno-arterial extracorporeal membranous oxygenation; ECMO: extracorporeal membranous oxygenation; LVAD: left ventricular assist devices; BiVAD: biventricular assist device.

After patients were listed on VA-ECMO, they had four possible mutually exclusive outcomes: OHT, VAD implantation, death, or survival off VA-ECMO, VAD or transplant. Therefore, after one-year from VA-ECMO, 129 (36%) patients underwent OHT without VAD bridging, 33 (9%) underwent VAD implantation, 125 (35%) patients died, and 68 (19%) patients survived free of OHT or VAD. (Figure 1). Dischargeable devices were used in 15/33, non-dischargeable in 7/33 and the type of VAD was unknown in 11 patients. Dischargeable devices were most commonly Heartmate II followed by HeartWare™ HVAD ™. (Table 1).

### 3.2. Waitlist Mortality

One hundred and twenty-six (126) patients died within one year from listing and prior to receiving OHT, accounting for waitlist mortality of 35.4%. Table 2 summarizes overall patients’ characteristics and the univariate predictors for waitlist mortality. Patients who did not survive to transplant were more likely to have higher BMI, diabetes mellitus, worse renal function, be on dialysis or on mechanical ventilation at the time of listing, and less likely to have non-ischemic cardiomyopathy or to have undergone VAD implantation during listing. A Cox proportional-hazards model, and multivariate analysis confirmed that factors associated with higher waitlist mortality included lack of VAD implantation (Hazard ratio HR 21.28, 95% confidence interval CI: 2.96–152.84, *p* = 0.002) and diabetes (HR 1.61, 95% CI: 1.08–2.40, *p* = 0.02). Conversely, non-ischemic cardiomyopathy was associated with lower waitlist mortality (HR 0.52, 95% CI: 0.31–0.87, *p* = 0.01).

Amongst the 33 patients who underwent VAD implantation, one patient died before transplantation at 12 months accounting for waitlist mortality of 3%. This group of patients was similar in most of their baseline characteristics at the time of listing including age, rate of mechanical ventilation and dialysis compared to the group of patients who did not undergo VAD implantation. On the other hand, the VAD group were more likely to have non-ischemic cardiomyopathy (48.5% vs. 25.2%), and less likely to be obese (6.1% vs. 25.2%) or have a history of prior organ transplant (3% vs. 31.1%). Patients who underwent VAD implantation had more days on the list (189 vs. 14), days in status 1A (37 vs. 5) and were more likely to undergo heart transplantation (75.8% vs. 40.1%) Table 3.

**Figure 2:**
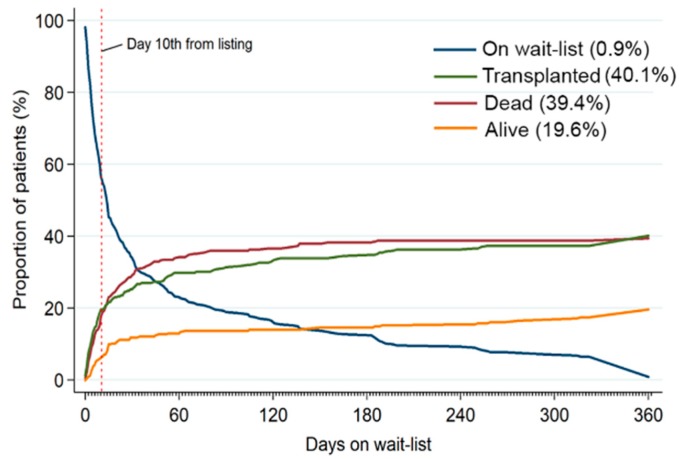
Competing outcomes of transplant, death, and survival show the chance for transplantation was highest in the first 10 days post-listing after which it markedly decreased. However, the mortality risk continued to stay high for 28 days post-listing. Therefore, VAD implantation should be considered in patients waiting for heart transplant >10 days on VA-ECMO. VAD: ventricular assist devices, VA-ECMO: veno-arterial extracorporeal membranous oxygenation.

Competing outcomes in patients who did not undergo VAD implantation showed that the chance for transplantation was highest in the first 10 days post-listing, after which it markedly decreased. The mortality risk was high initially but continued to stay the same up to 28 days post-listing. Therefore, VAD implantation should be considered if patients wait for a transplant for more than 10 days. (Figure 2).

### 3.3. Post-Transplant Survival

Amongst the 355 patients listed on VA-ECMO, 154 (43%) eventually received a heart transplant. One-year post-transplant survival was 62.3% (96/154). Amongst the 33 patients who underwent VAD implantation from VA-ECMO, 25 (75%) were eventually transplanted and eight (25%) remained on VAD support. The non-VAD group had similar age, comorbidities, rate of dialysis and mechanical ventilation at the time of listing compared to the VAD group. The VAD group were less likely to have a history of organ transplant (4% vs. 24.8%, *p* = 0.02). Table 4.

**Figure 3:**
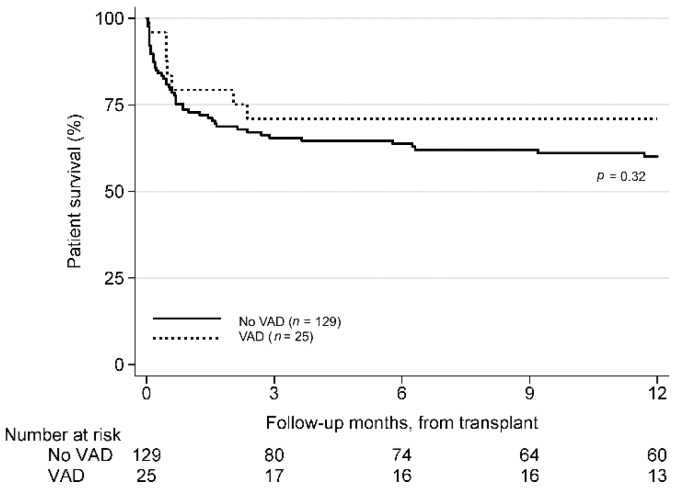
One year post-transplant survival. Kaplan Meier curves for one year post-listing survival is presented comparing patients supported with VAD during listing against those without. No difference in survival is seen between the two groups.

The VAD group had higher although not statistically significantly different one-year post-transplant survival (72.0% vs. 60.5%, *p* = 0.323) when compared to the non-VAD group (Figure 3).

Table 5 summarizes the univariate and multivariate predictors of one-year post-transplant mortality of the overall cohort. Independent predictors of post-transplant mortality included PRA class I ≥ 20% (HR 2.36, 95% CI: 1.29 to 4.29, *p* = 0.005), history of smoking (HR 1.92, 95% CI: 1.06 to 3.48, *p* = 0.032), higher serum creatinine (HR 1.76, 95% CI: 1.26 to 2.47, *p* = 0.001) and higher total bilirubin level (HR 1.05, 95% CI: 1.01 to 1.102.47, *p* = 0.022) at time of transplant. Conversely, age, race, BMI, donor-recipient, and gender mismatch were not associated with higher mortality.

## 4. Discussion

In this analysis of the UNOS database of patients listed for OHT with VA-ECMO, we found the following three primary findings: (1) use of VAD implantation after listing with VA-ECMO was low (10%), (2) survival of VAD patients when transplanted was similar to the non-VAD group, and (3) PRA class I ≥ 20, smoking, serum creatinine, and total bilirubin at the time of transplant were predictors of one-year post-transplant mortality after being listed with VA ECMO.

Patients with refractory cardiogenic shock and multi-organ failure have high mortality despite any intervention. Advances in the VA-ECMO circuit and increasing experience has made VA-ECMO viable temporary support in these patients. Despite these advances, the complication rate of VA-ECMO, such as major bleeding and neurological complications, are high and increase with the duration of VA-ECMO [4,11]. In patients listed with VA-ECMO for OHT these complications can be catastrophic as they could preclude placement of a more durable device or OHT. Furthermore, while VA-ECMO provides adequate hemodynamic support, it does not provide adequate left ventricle unloading in some patients due to increased left ventricular (LV) afterload which can result in thrombus formation and pulmonary edema in about 20% [12,13]. Therefore, there is a narrow window of opportunity in patients on VA-ECMO support between recovery from multi-organ failure to development of VA ECMO related complications during which a durable intervention (OHT and VAD) can be life-saving [13]. Colvin et al. recently reported that waitlist mortality is highest in patients with VA-ECMO compared to other patients on the waitlist (41.6% vs. 5.9%) [4]. We found a similar observation as waitlist mortality in our study was 35.4%. Yet there is insufficient data regarding factors associated with increased mortality after being listed on VA-ECMO. Our study findings suggest that patients who received a VAD from VA-ECMO had longer days on the waitlist (189 vs. 14 days) and had decreased waitlist mortality (3% vs. 38%). Two prior single center studies evaluated the short-term outcomes of transition from VA-ECMO to VAD support. In the first study, amongst 73 patients on VA-ECMO, 11 were crossed over to any durable or total artificial heart (TAH) and had improved 30-days survival (72% vs. 18%) [14]. In the second study, 16 patients who were bridged to VAD had better survival when compared to those who were not [15]. However, in the latter study, all patients who survived VAD bridge subsequently underwent OHT. Similarly, in this analysis the majority in the VAD group (75.7%) were also bridged to a heart transplant but 25% who did not receive a heart transplant had a survival of 87.5%.

Collectively, these observations combined with ours can guide centers who are considering a durable device bridge from VA ECMO in patients who are considered to be candidates for one. Although the simulated modeling of the proposed allocation system shortens waitlist time and thus theoretically can improve waitlist mortality, our data suggests that centers should consider VAD implantation in patients after VA-ECMO if they are deemed candidates for one as our data did not study factors associated with high-risk VAD implantations, such as small left ventricle size, large burden of ventricular tachycardia, and peripheral vascular disease.

## 5. Survival after OHT from VA- ECMO

Survival after OHT, when bridged with VA-ECMO, is dismal compared to patients bridged with assist devices [16]. A recent study found that patients with ongoing renal dysfunction and mechanical ventilation are particularly at higher risk of mortality after OHT from VA-ECMO [17]. There are mixed outcomes from single center experiences looking at sequential devices prior to OHT. One study suggested that sequential devices may lead to worse outcomes compared with an upfront LVAD bridge to OHT strategy [18]. However, this study did not specifically look at VA-ECMO bridge to VAD prior to transplant. Other single center experiences have suggested that a bridge to bridge strategy from VA-ECMO may lead to equivalent outcomes compared to a direct bridge to transplantation [13,15]. These findings were from smaller cohorts (<20 patients) in France and Taiwan, wherein the institutional practices and allocation criteria were different.

To our knowledge, our analysis is based on the largest sample size to define one-year post-transplant survival after listing on VA-ECMO and the impact of VAD on this outcome. Compared to the current national one-year post-OHT survival of 89.6% [19], a direct transplant from VA-ECMO was associated with a much lower survival rate of 60.5%. In comparison, post-OHT survival with the use of sequential VAD after VA-ECMO was higher at 72% (albeit not statistically significantly different) which is still lower than current LVAD bridge-to-transplant survival [4]. Although the use of non-dischargeable percutaneously implanted VADs is on the rise, there is a paucity of data that defines post-transplant survival with the use of these temporary devices. In our study, seven out of 33 patients were supported by surgically placed non-dischargeable VADs. The small sample size in addition to not knowing the type of VAD in 11 patients in our study, precluded analyzing post heart transplantation survival by device type.

Lastly, we found that patients with high PRA I ≥ 20% and similar to prior studies end-organ dysfunction (liver and kidney) were at increased risk of mortality post-transplant. Presence of renal failure has been shown to be a risk factor for post-transplant survival in other studies [17,20]. Sensitized patients on VA-ECMO face a particular challenge since they do not have the luxury of time to wait for a completely immunocompatible donor, which increases the risk of rejection in the post-operative period and decreased survival.

## 6. Limitations

Our study has several limitations. First, as this is a retrospective analysis of the UNOS database, we did not have specific hemodynamic data, device type in 11 patients, and information regarding the cause of death. Second, various VADs were used to support patients after VA-ECMO. The clinical course and outcomes can be different for these different devices which we were not able to characterize due to the small sample size of this cohort. Similarly, percutaneous short-term devices were not used and therefore were not examined which is perhaps different from current practice at many institutions. Third, we did not account for time on VA-ECMO support prior to listing or the time between VA-ECMO support and VAD implantation as this data was not available. Fourth, we did not have information regarding the duration of heart failure prior to VA-ECMO, the selection process for VAD implantation or information regarding patient status at the time of VAD implantation. Fifth, the loss of statistical significance in post-transplant survival between patients who were transplanted from VAD vs. directly from VA-ECMO could be related to the small number of patients in our analysis. A significant difference may have been detected in a larger cohort. Lastly, use of VA-ECMO as a temporary support device and outcomes depend on institutional protocols and experience and we did not have this type of granular data for analysis.

## 7. Conclusions

In the era of increasing patients’ medical complexity and limited organ availability, our study from the UNOS database shows that durable VAD implantation from VA-ECMO bridge is associated with a higher number of days on the waitlist. A bridge from VA-ECMO to such devices and then to transplantation compared to a VA-ECMO direct bridge to transplantation led to a higher, although not significantly different, one-year post-transplant survival.

## Figures and Tables

**Table 1:** Ventricular assist devices types.

VAD Brand	Non-Transplanted	Transplanted	Total
**HeartMate II**	2	6	8
**Heartware HVAD**	0	5	5
**Abiomed BVS 5000**	0	2	2
**HeartMate XVE**	0	1	1
**Impella Recover 2.5**	1	0	1
**Levitronix Centrimag**	0	1	1
**Syncardia CardioWest**	0	1	1
**Thoratec**	1	2	3
**Unknown**	4	7	11
**Total**	8	25	33

VAD: ventricular assist device; HVAD: HeartWare ventricular assist device; BVS: bioresorbable vascular scaffold.

**Table 2:** Baseline characteristics and univariate predictors of waitlist mortality (death on the waitlist).

	Total (*n* = 355)	Alive or Transplanted (*n* = 229)	Dead (*n* = 126)	Unadjusted HR (95% CI)	*p*-Value
**Demographics and past medical history**					
Age	50 (38–60)	50 (37–59)	52.5 (41–60)	1.01 (1.00–1.02)	0.16
Male	236 (66.5)	151 (65.9)	85 (67.5)	1.01 (0.70–1.47)	0.95
Non-white	97 (27.3)	56 (24.5)	41 (32.5)	1.41 (0.97–2.05)	0.07
BMI	26.3 (23.8–30.0)	25.5 (23.0–29.2)	27.7 (24.6–32.6)	1.04 (1.01–1.07)	0.01
Non-O ABO blood type	209 (58.9)	143 (62.4)	66 (52.4)	0.79 (0.56–1.12)	0.19
Smoking	123 (34.6)	74 (32.3)	49 (38.9)	1.31 (0.91–1.87)	0.14
Diabetes	81 (22.8)	40 (17.5)	41 (32.5)	1.94 (1.33–2.82)	0.001
Cardiac surgery	160 (45.1)	101 (44.1)	59 (46.8)	1.16 (0.82–1.66)	0.40
NICMP	97 (27.3)	77 (33.6)	20 (15.9)	0.42 (0.26–0.68)	<0.001
**Characteristics at time of listing**					
Mechanical Ventilation	157 (44.2)	92 (40.2)	65 (51.6)	1.54 (1.09–2.19)	0.02
Dialysis	42 (11.8)	21 (9.2)	21 (16.7)	1.80 (1.12–2.87)	0.02
eGFR < 60	181 (51)	107 (46.7)	74 (58.7)	1.50 (1.05–2.14)	0.03
Inotrope	146 (41.1)	94 (41.0)	52 (41.3)	1.01 (0.71–1.44)	0.97
Mean PAP	28.0 (22.0–37.0)	28.0 (21.5–38.5)	31.0 (22.5–35.0)	1.00 (0.98–1.02)	0.93
Mean PCWP	20.0 (13.0–26.0)	20.0 (14.0–27.0)	20.0 (12.0–24.0)	0.98 (0.95–1.01)	0.21
Cardiac output	3.9 (2.9–4.8)	3.9 (2.9–4.7)	3.7 (2.8–4.9)	1.00 (0.84–1.18)	0.96
No VAD implant	332 (90.7)	197 (86.0)	125 (99.2)	19.26 (2.69 -137.90)	0.003

BMI: body mass index, NICMP: non-ischemic cardiomyopathy, PAP: pulmonary artery pressure, PCWP: pulmonary capillary wedge pressure, VAD: ventricular assist devices. eGFR: estimated glomerular filtration rate, HR: hazard ratio. Values expressed as median (IQ) or number (%).

**Table 3:** Demographic and clinical characteristics in patients who had VAD implantation during listing.

	No VAD (*n* = 322)	VAD (*n* = 33)	*p*-Value
Age	51 (37–60)	48 (42–59)	0.85
Male	209 (64.9)	27 (81.8)	0.05
Non-AA	52 (16.2)	6 (18.2)	0.76
Obese (BMI ≥ 30)	81 (25.2)	2 (6.1)	0.01
Non-O ABO	190 (59.0)	19 (57.6)	0.87
Smoking	114 (35.4)	9 (27.3)	0.35
Diabetes	77 (23.9)	4 (12.1)	0.12
Cardiac surgery	148 (46.0)	12 (36.4)	0.29
Prior transplant	100 (31.1)	1 (3.0)	0.001
NICMP	81 (25.2)	16 (48.5)	0.004
Ventilation *	146 (45.3)	11 (33.3)	0.19
Dialysis *	38 (11.8)	4 (12.1)	0.96
Inotrope *	134 (41.6)	12 (36.4)	0.56
Days on listing	14 (5–52)	189 (77–283)	<0.001
Days in 1A status	5 (2–11)	37 (19–58)	<0.001
Transplant after listing	129 (40.1)	25 (75.8)	<0.001

AA: African American, BMI: body mass index, NICMP: non-ischemic cardiomyopathy, VAD: ventricular assist devices. * at time of listing. Values expressed as median (IQ) or number (%).

**Table 4:** Demographic and clinical characteristics in patients who were transplanted with vs. without VAD implantation during listing.

	No VAD Prior to OHT (*n* = 129)	VAD Prior to OHT (*n* = 25)	*p*-Value
Age	50 (37–60)	55 (45–63)	0.13
Male	83 (64.3)	21 (84.0)	0.06
Non-AA	111 (86.1)	22 (88.0)	0.79
Obese (BMI ≥ 30)	25 (19.4)	4 (16.0)	0.69
Non-O ABO	81 (62.8)	159 (60.0)	0.79
Smoking	38 (29.5)	7 (28.0)	0.88
Diabetes	24 (18.6)	4 (16.0)	0.76
Cardiac surgery	68 (52.7)	9 (36.0)	0.13
Prior transplant	32 (24.8)	1 (4.0)	0.02
NICMP	40 (31.0)	12 (48.0)	0.10
Ventilation *	47 (36.4)	5 (20.0)	0.11
Dialysis *	14 (10.9)	3 (12.0)	0.87
Inotrope *	55 (42.6)	8 (32.0)	0.32
Days on listing	12 (4–57)	152 (66–259)	<0.001
Days in 1A status	5 (2–20)	35 (22–49)	<0.001

AA: African American, BMI: body mass index, NICMP: non-ischemic cardiomyopathy, VAD: ventricular assist devices. OHT: orthotopic heart transplantation. * at time of listing. Values expressed as median (IQ) or number (%).

**Table 5:** Univariate and multivariate predictors of post-transplant mortality.

Variable	Total (*n* = 154)	Post-Transplant Mortality			
		Univariate		Multivariate	
		HR (95% CI)	*p*-Value	HR (95% CI)	*p*-Value
**Recipient’s Demographics and Past Medical History**					
Age	50 (38–60)	1.01 (0.99–1.02)	0.48	1.01 (0.99–1.03)	0.297
Male	104 (67.5)	0.78 (0.45–1.34)	0.37		
Non-White	32 (20.8)	2.42 (1.40–4.19)	0.002	1.60 (0.86–2.99)	0.140
BMI *	25.3 (22.2–28.4)	1.06 (1.01–1.11)	0.02	1.03 (0.98–1.09)	0.218
Non-O ABO	96 (62.3)	1.54 (0.87–2.72)	0.14		
Ischemic time ≥ 4	33 (22.3)	1.17 (0.62–2.19)	0.63		
PRA I ≥ 20%	34 (22.1)	2.13 (1.21–3.74)	0.008	2.36 (1.29–4.29)	0.005
PRA II ≥ 20%	34 (22.1)	0.90 (0.47–1.74)	0.76		
Heart re-transplant	33 (21.4)	1.59 (0.88–2.87)	0.13		
Smoking history	45 (29.2)	1.71 (1.00–2.92)	0.051	1.92 (1.06–3.48)	0.032
Diabetes	28 (18.2)	1.48 (0.81–2.72)	0.20		
Creatinine *	1.2 (0.9–1.6)	1.64 (1.21–2.22)	0.001	1.76 (1.26–2.47)	0.001
Total bilirubin *	1.2 (0.6–2.0)	1.05 (1.01–1.09)	0.01	1.05 (1.0–1.10)	0.022
Mean PAP *	26.0 (20.0–35.0)	1.00 (0.97–1.03)	0.98		
Mean PCWP *	17.0 (13.0–26.0)	1.00 (0.96–1.04)	0.99		
Cardiac output *	4.0 (3.1–5.2)	1.14 (0.94–1.39)	0.19		
High risk CMV	39 (27.7)	0.48 (0.20–1.12)	0.09		
Dialysis	29 (19.0)	1.43 (0.77–2.66)	0.26		
Gender mismatch	49 (31.8)	2.33 (1.38–3.95)	0.002	1.64 (0.94–2.87)	0.080
NICMP	52 (33.8)	0.62 (0.34–1.14)	0.13		
No VAD	129 (83.8)	0.67 (0.31–1.49)	0.33	1.85 (0.81–4.21)	0.145
**Donor’s Demographics and Past Medical History**					
Age ≥ 50	20 (13.0)	0.69 (0.27–1.73)	0.43		
BMI ≥ 30	31 (20.1)	0.69 (0.34–1.41)	0.31		
Smoking history	27 (17.8)	0.92 (0.44–1.95)	0.83		
Diabetes	5 (3.3)	0.70 (0.10–5.04)	0.72		
Hypertension	21 (13.7)	0.64 (0.26–1.60)	0.34		

BMI: body mass index, PRA: panel reactive antibodies, PAP: pulmonary artery pressure, PCWP: pulmonary capillary wedge pressure, CVM: cytomegalovirus, NICMP: non-ischemic cardiomyopathy, VAD: ventricular assist devices. * at time of transplant. Values expressed as median (IQ) or number (%).
